# Not All Microbiomes Reflect Chronic Pain: Evidence from the Urinary Tract in a Case–Control Study

**DOI:** 10.3390/jcm15134931

**Published:** 2026-06-25

**Authors:** Lisa Goudman, Maarten Moens

**Affiliations:** 1 STIMULUS Research Group, Vrije Universiteit Brussel, Laarbeeklaan 103, 1090 Brussels, Belgium; 2 Department of Neurosurgery, Universitair Ziekenhuis Brussel, Laarbeeklaan 101, 1090 Brussels, Belgium; 3 Center for Neurosciences (C4N), Vrije Universiteit Brussel, Laarbeeklaan 103, 1090 Brussels, Belgium; 4 Research Foundation—Flanders (FWO), 1090 Brussels, Belgium; 5 Pain in Motion Research Group (PAIN), Department of Physiotherapy, Human Physiology and Anatomy, Faculty of Physical Education and Physiotherapy, Vrije Universiteit Brussel, Laarbeeklaan 103, 1090 Brussels, Belgium; 6 Department of Radiology, Universitair Ziekenhuis Brussel, Laarbeeklaan 101, 1090 Brussels, Belgium

**Keywords:** chronic pain, urinary microbiome, 16S rRNA gene sequencing, microbial diversity, pain mechanisms

## Abstract

**Background/Objectives**: Chronic pain is increasingly conceptualized as a systemic condition characterized by central sensitization, autonomic dysregulation, and persistent neuroimmune and neuroendocrine alterations. These systemic changes have been linked to microbial dysbiosis, most prominently within the gut microbiome. In contrast, the relevance of the urinary microbiome outside primary urological disease remains poorly understood, particularly in non-urological chronic pain conditions. The objective of this study was to determine whether patients with chronic low back pain exhibit differences in urinary microbial diversity, community composition, or taxon-specific abundance compared with pain-free controls. **Methods**: In this age- and sex-matched case–control study, midstream urine samples were collected from ten patients with chronic low back pain and ten pain-free controls and analyzed using 16S rRNA gene sequencing (V4 region). Sequence data were processed using nf-core/ampliseq and DADA2. Alpha diversity, beta diversity, and differential abundance were assessed using depth-adjusted models, compositional and phylogenetically informed distance metrics, and ANCOM-BC2, with multiple sensitivity analyses to account for the low-biomass nature of urinary microbiome data. **Results**: After accounting for sequencing depth, no significant differences in alpha diversity were observed between patients and controls for any metric. Beta diversity analyses revealed no significant differences in overall community composition between groups across all distance measures, and dispersion was comparable between groups. Differential abundance analysis did not identify any bacterial taxa that differed significantly between patients and controls after correction for multiple testing. **Conclusions**: In this cohort, chronic low back pain was not associated with detectable alterations in the urinary microbiome. These findings suggest that, unlike the gut microbiome, urinary microbial communities may be relatively stable in the context of non-urological chronic pain, highlighting the importance of phenotype specificity and multidimensional approaches in microbiome-based pain research.

## 1. Introduction

Chronic pain conditions are increasingly conceptualized as disorders characterized by central sensitization, consisting of sustained hyperexcitability of nociceptive pathways, impaired inhibitory control, and activity-dependent amplification of sensory input within spinal and supraspinal circuits [1,2,3,4,5]. These neural changes are accompanied by dysregulation of the autonomic nervous system, reflecting impaired central autonomic integration, as well as persistent neuroimmune activation involving both peripheral immune mediators and central glial signalling [6,7,8,9]. Alterations in neuroendocrine regulation, particularly within the hypothalamic–pituitary–adrenal axis, further suggest prolonged engagement of stress-responsive physiological systems [10,11]. Together, these findings support a systems-level view of chronic pain with the potential to influence multiple organ systems beyond the primary pain location [12].

In the urinary tract, microbial community composition is thought to be shaped by a combination of host-derived ecological pressures, including local immune activity, epithelial barrier function, urine chemistry, and neurogenic regulation of lower urinary tract function [13,14,15]. Urothelial cells are increasingly recognized as active participants in host–microbe interactions, capable of responding to neural and inflammatory signals and releasing mediators that influence the local microenvironment [14,16]. The urinary microbiome may therefore be considered part of a local host–microbe ecosystem that interacts with mucosal and immune pathways contributing to urinary tract homeostasis [17]. In urological pain conditions, particularly interstitial cystitis/bladder pain syndrome, studies have reported altered urinary microbiota as well as local or systemic immune activation, suggesting that host–microbe–immune interactions may be relevant to nociceptive signalling in conditions directly involving the lower urinary tract [18,19]. Cytokines and chemokines detected in urine, serum, or bladder tissue of patients with bladder pain syndromes, including IL-1β, IL-6, TNF-α, IL-8/CXCL8, CXCL1, CXCL10, and CCL2/MCP-1, further support a potential role for immune mediators in urothelial inflammation and pain signalling, although their direct relationship with urinary microbial community states remains incompletely understood [18,19,20,21]. Beyond local inflammation, neurogenic regulation of bladder function and proposed bladder–gut–brain axis interactions provide a conceptual framework through which urinary tract signals may interact with autonomic, immune, and central pain-processing pathways [22,23]. Consequently, systemic alterations in autonomic, immune, and neuroendocrine signalling, as observed in chronic pain, may plausibly influence urinary tract physiology and thereby contribute to variation in urinary microbial community structure, even in the absence of overt urinary tract pathology [13,24].

At the same time, the urobiome, or the microbiota of the urinary tract, comprises the microbial community of the kidneys, ureters, bladder and urethra, although most human studies infer this primarily from urine sampling [25,26]. Compared to the gut, the urobiome has a relatively low microbial biomass (below 10^5^ colony units per mL of urine versus 10^11^ bacteria per gram of feces [27,28]), which increases vulnerability to external contamination and complicates DNA extraction and interpretation [29]. As a result, observed differences in urinary microbial composition must be interpreted cautiously and within the context of rigorous contamination control and appropriate analytical frameworks [30,31]. Despite these challenges, accumulating evidence supports the biological relevance of urinary microbial communities, underscoring the importance of methodologically robust studies in populations not traditionally considered at risk for urological disease [32].

To date, investigations of the urinary microbiome have focused predominantly on patients with interstitial cystitis/bladder pain syndrome and chronic prostatitis/chronic pelvic pain syndrome [33,34,35,36], where microbiome-targeted interventions, including probiotics aimed at gut microbiota modulation, have been explored as potential therapeutic strategies in chronic bacterial prostatitis [37]. While alterations in urinary microbial community composition have been reported in these lower urinary tract disorders, the urinary microbiome has not been systematically examined in non-urological chronic pain cohorts. Importantly, chronic low back pain is frequently associated with widespread sensory, autonomic, and somatic symptoms, supporting its classification as a systemic pain condition rather than a localized musculoskeletal disorder [6,38]. However, whether such systemic pain-related alterations are sufficiently reflected in the urinary microbial ecosystem remains unknown. Within this framework, the urinary microbiome may serve as a sensitive biological readout of host physiological state, rather than a primary driver of pathology. Addressing this gap, the present study examines whether individuals with chronic low back pain exhibit differences in urinary microbial community structure compared with pain-free controls.

## 2. Materials and Methods

### 2.1. Study Design

This sex-and age-matched case–control study was designed to assess urinary microbiota composition in low back pain patients compared to pain-free controls. The study was conducted according to the revised Declaration of Helsinki (1998). The study protocol was approved by the ethics committee of VUB/Universitair Ziekenhuis Brussel (B.U.N. 1432024000293) on 18 December 2024. All participants provided written informed consent before enrolment in the study.

The primary objective was to compare urinary microbial community composition between patients with chronic low back pain and pain-free controls. Secondary objectives included assessment of alpha diversity, beta diversity, and differential taxonomic abundance between groups.

No formal a priori power calculation was performed because reliable effect-size estimates for urinary microbiome differences between patients with chronic low back pain and pain-free controls were not available at study inception. We therefore designed this study as exploratory and pragmatically aimed to enrol 10 participants per group, balancing feasibility, sequencing costs, and recruitment constraints in this clinically selected population. Accordingly, the study should be regarded as hypothesis-generating.

### 2.2. Study Participants

Patients were recruited from the Radiology department at UZ Brussel. Pain-free controls were selected by convenience sampling by asking contacts of the study team members to participate in this study. Controls were not household controls and were not cohabiting with patients. Both groups consisted of 10 participants, age-and sex matched with a range of 5 years for age matching.

Inclusion criteria for patients were chronic low back pain of at least 3 months’ duration and clinical classification of nociceptive facet-mediated pain based on routine clinical assessment supported by positive scintigraphy at a corresponding lumbar facet joint. This classification reflected presumed peripheral nociceptive input from facet joint pathology, but did not necessarily indicate a specific inflammatory mechanism. Although facet joint degeneration may be accompanied by local inflammatory or metabolically active changes, inflammatory activity was not directly assessed in this study. Pain-free controls were defined as individuals without acute or chronic pain conditions.

Patients and pain-free controls were not eligible if they received antibiotics or probiotic supplements for at least one month prior to sampling [39]. Additionally, patients with an active psychiatric disorder, pregnant females or known active urinary tract infection were excluded. Use of analgesic medication, including non-steroidal anti-inflammatory drugs and opioids, was permitted.

### 2.3. Clinical Assessment

Current pain intensity was measured with the Visual Analogue Scale (VAS) (ranging from 0 (no pain) to 100 (maximal pain)) in electronic format. No clinically relevant difference exists between the traditional paper-based VAS assessment and VAS scores obtained from laptop computer- and mobile phone-based platforms for adults [40]. No additional standardized pain phenotype questionnaires (e.g., neuropathic pain or central sensitization screening tools) or quality of life questionnaires were systematically applied as part of routine clinical care.

### 2.4. Urine Collection and Storage

Urine samples were collected through midstream urine samples, with the first millilitres discarded as they may be contaminated by bacteria from the skin. All participants were provided with a self-sampling kit to collect their urine. Instructions on the sampling technique were provided before sampling, and a small folder with this information was provided to all participants. The samples were stored at UZ Brussel Biobank as soon as possible after urine collection at −80 °C until further analysis. Samples remained frozen until processing and were never thawed before.

### 2.5. DNA Extraction and Library Preparation

Microbial DNA was extracted from 230 µL of the urine sample collected in eNAT tubes. Prior to extraction, 20 µL of ZymoBIOMICS™ Spike-in Control I (*Imtechella halotolerans*, *Allobacillus halotolerans*; Zymo Research, Irvine, CA, USA) was added, and samples were transferred to ZR BashingBead™ lysis tubes (Zymo Research) to facilitate mechanical disruption using a bead-beating step.

Subsequently, 300 µL of lysate was processed for DNA extraction using the Maxwell^®^ RSC Fecal Microbiome DNA Kit (Promega, Madison, WI, USA) according to the manufacturer’s instructions.

DNA concentration was quantified using the QuantiFluor™ One dsDNA System (Promega) on a Qubit fluorometer (Thermo Fisher Scientific, Waltham, MA, USA).

### 2.6. 16S rRNA Gene Sequencing

The V4 region of the bacterial 16S rRNA gene was amplified using the 515F (GTGCCAGCMGCCGCGGTAA) and 806R (GGACTACHVGGGTWTCTAAT) primer pair. Sequencing was performed by BRIGHTCore (UZ Brussel, Brussels, Belgium) on an Illumina MiSeq platform (Illumina, San Diego, CA, USA), using paired-end sequencing (2 × 300 bp), yielding a median sequencing depth of approximately 60,000 reads per sample.

### 2.7. Bioinformatics and Sequence Processing

Sequence data was processed using nf-core/ampliseq version 2.14.0 [41] of the nf-core collection of workflows [42], utilizing reproducible software environments from the Bioconda [43] and Biocontainers [44] projects. Read quality was evaluated with FastQC and summarized with MultiQC [45]. Cutadapt [46] was used to remove primer sequences. Reads lacking the expected primer sequence were discarded, as primer artifacts can introduce spurious variation, especially with degenerate primers. On average, 78.2% of reads passed primer trimming.

Primer-free reads were processed using DADA2 [47] to remove PhiX contamination, trim reads (forward to 200 bp and reverse to 140 bp), discard reads with >2 expected errors, correct sequencing errors, merge paired-end reads, and remove PCR chimeras. This procedure resulted in 457 amplicon sequencing variants (ASVs) across all samples. Between 85.48% and 96.75% of reads per sample (average: 94.7%) were retained after DADA2 processing.

Taxonomic classification was performed by DADA2 against the ‘Silva 138.2 prokaryotic SSU’ database [48]. ASVs lacking classification at a given rank were retained and labelled as “unclassified” at their highest resolved level rather than being pooled into a single undifferentiated category, ensuring that unclassified taxa contributed appropriately to diversity analyses. One ASV classified as a chloroplast or mitochondrial sequence was removed. Of the inferred amplicon sequence variants (ASVs), 99.12% of ASVs were classified at the phylum level, 93% at the family level, and 87.09% at the genus level, while 45.95% could be resolved to the species level.

ASV sequences, abundance and taxonomy were then loaded into QIIME2 [49] to generate standard exploratory outputs (barplots, alpha rarefaction curves) and to verify that sequencing depth was sufficient. ANCOM-BC was also run within QIIME2 for exploratory purposes, but all differential-abundance results reported in this manuscript are based on ANCOM-BC2 models fitted in R.

### 2.8. Statistical Analysis

Microbiome analyses were performed in R Studio (version 2022.07.2, Posit Software PBC, Boston, MA, USA). As a basic quality-control measure, samples with fewer than 1000 total reads were excluded, resulting in the removal of one sample and a minimum sequencing depth of 1161 reads. ASVs with fewer than 10 total counts across all samples or present in <5% of samples were removed. After applying all filtering steps, the final phyloseq object contained 361 ASVs across all samples. Descriptive statistics are presented as mean ± standard deviation (SD) or median with first and third quartiles.

#### 2.8.1. Alpha Diversity

Alpha diversity was assessed using entropy-based indices (Shannon, Simpson, inverse Simpson), richness-based metrics (observed ASV richness and Chao1), and Pielou’s evenness (calculated as the Shannon index divided by the natural logarithm of observed ASV richness). Before calculating alpha diversity metrics on the filtered, non-rarefied count table, correlations with sequencing depth were evaluated. Sequencing depth varied widely across urine samples and showed significant or borderline associations with entropy-based diversity indices and richness-based metrics, whereas evenness did not show a significant association with sequencing depth. Accordingly, entropy-based diversity indices and richness-based metrics were primarily analyzed using linear models with population as the main predictor and log-transformed sequencing depth included as a covariate to account for depth-related bias. Evenness was analyzed using linear models with population as the sole predictor. As a sensitivity analysis, alpha diversity comparisons were repeated after rarefaction to an even sequencing depth. To avoid excessive loss of sequencing information, samples were rarefied to the first quartile of sequencing depth (18,771 reads), and samples with lower sequencing depth were excluded from this analysis. Results from rarefied and depth-adjusted analyses were compared to assess the robustness of findings.

#### 2.8.2. Beta-Diversity

Between-sample community dissimilarity was assessed using multiple complementary distance metrics. Bray–Curtis dissimilarity was calculated on relative-abundance ASV tables to assess ecological differences in community composition. To account for the compositional nature of microbiome data, a centred log-ratio (CLR) transformation was applied to ASV counts (pseudocount = 1), and Euclidean distances in CLR space (Aitchison distance) were calculated. In addition, phylogeny-informed community differences were evaluated using weighted UniFrac distances, based on the available rooted phylogenetic tree. Ordinations were visualized using principal coordinates analysis (PCoA). Group-level differences between controls and patients were tested using permutational multivariate analysis of variance (PERMANOVA; adonis2) with 9999 permutations. Given the wide range of sequencing depths across urine samples, sensitivity analyses additionally included log-transformed sequencing depth as a covariate. Homogeneity of multivariate dispersions was assessed using PERMDISP (betadisper) to evaluate whether observed differences reflected shifts in centroid location rather than differences in dispersion.

#### 2.8.3. Differential Abundance

Differential abundance testing was performed using ANCOM-BC2, which accounts for the compositional nature of microbiome count data and differences in sampling fractions across samples. Analyses were performed on the filtered, non-rarefied ASV count table. Taxa were tested primarily at the genus level, and a sensitivity analysis was performed at the ASV level. Population (control vs. patient) was included as the fixed effect in the model, and no random effects were specified due to the cross-sectional design. Taxa present in fewer than 10% of samples were excluded prior to analysis. *p*-values were adjusted for multiple testing using the Benjamini–Hochberg false discovery rate procedure. Structural zeros and robustness filtering were enabled using default ANCOM-BC2 settings.

### 2.9. Contamination Control and Quality Filtering

Given the low-biomass nature of urine samples, contamination control and technical noise reduction were considered throughout laboratory processing and downstream analysis. One extraction blank containing ZymoBIOMICS Spike-in Control I was included and sequenced as NTC_urine, with results reflecting the expected presence of the spike-in control. One PCR no-template control consisting of nuclease-free water was also included during library preparation, but had a non-measurable DNA concentration, 0 ng/µL, and was therefore not sequenced. Reagent contamination was not assessed using a separate formal prevalence- or frequency-based contaminant analysis. No formal decontamination algorithm, such as decontam, was applied because the available extraction control contained spike-in DNA and therefore did not represent a true blank. Applying prevalence-based filtering in this context could lead to overcorrection and removal of genuine low-abundance urinary taxa. The results were therefore interpreted cautiously in light of the low-biomass sample type.

Spike-in-derived ASVs were identified based on taxonomic assignment and prevalence across samples and controls. ASVs assigned to the spike-in genus *Imtechella* were removed prior to downstream analyses. Removal of *Imtechella* ASVs eliminated a median of 2200 (Q1–Q3: 107.5–5186.2) reads per sample. Removal of the low-prevalence spike-in genus *Allobacillus* was evaluated in a sensitivity analysis and did not materially affect library sizes or downstream results. All analyses were therefore performed on data with *Imtechella* removed only.

## 3. Results

### 3.1. Descriptives

In both groups, seven females and three males were included. The median age in the pain-free group was 57.5 (Q1–Q3: 48.5–69) years, and in the patient group, 57 (Q1–Q3: 47–71) years (Table 1).

**Figure 1:**
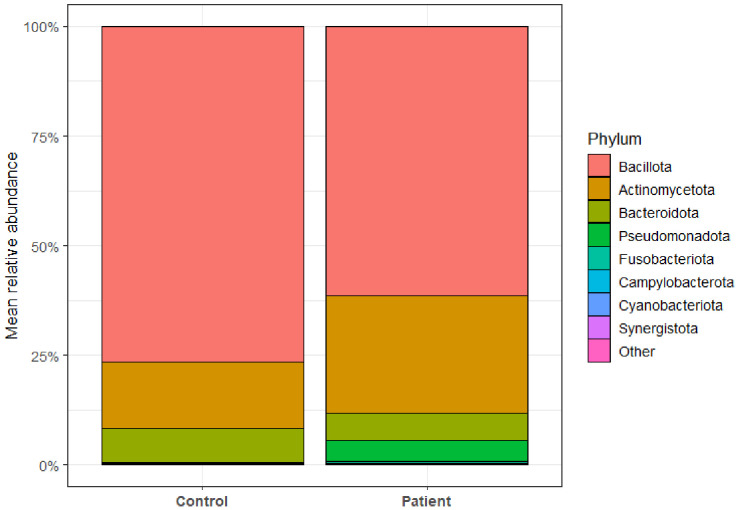
Phylum-level composition of the urinary microbiome in controls and patients. Bars represent mean relative abundance per group (top phyla shown; remaining phyla collapsed into “Other”).

The median pain intensity score was 72 (Q1–Q3: 54–79.25). Urine samples were collected for every participant, indicating no missing data. To provide an overview of microbial community composition, the relative abundance of phyla across participants and groups is shown in Figure 1.

### 3.2. Alpha Diversity

Sequencing depth across urine samples ranged from 1156 to 218,911 reads per sample (median: 66,149). Correlation analyses revealed moderate to strong associations between sequencing depth and entropy-based diversity indices (Simpson and inverse Simpson) as well as richness-based metrics (observed richness and Chao1), while Shannon diversity showed a borderline association with sequencing depth. In contrast, Pielou’s evenness did not show a significant association with sequencing depth.

**Figure 2:**
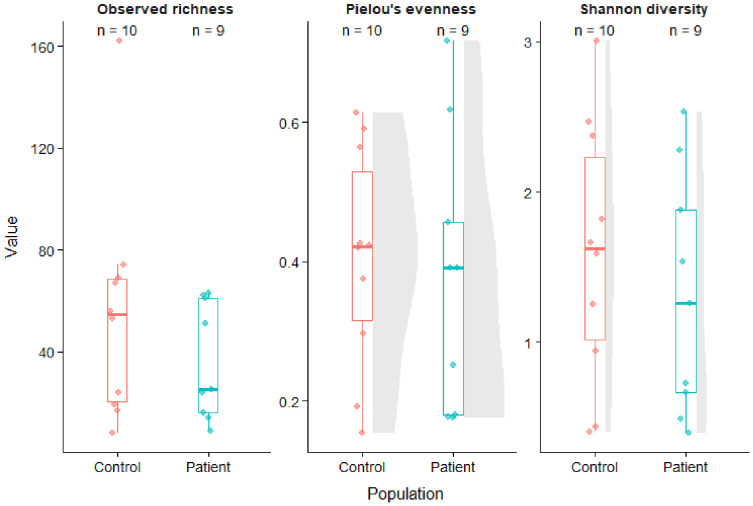
Alpha diversity of the urinary microbiome across populations. Raincloud plots show Shannon diversity, Pielou’s evenness, and observed ASV richness calculated on the filtered ASV table. Boxes indicate median and interquartile range; points represent individual samples; half-violins represent the distribution. Statistical comparisons were performed using linear models; Shannon and richness were adjusted for log-transformed sequencing depth, whereas evenness was analyzed without depth adjustment.

After adjusting for sequencing depth using linear models with log-transformed library size as a covariate, no significant differences in alpha diversity were observed between populations for Shannon diversity (F(1,16) = 0.53, *p* = 0.48), Simpson index (F(1,16) = 0.67, *p* = 0.42), or inverse Simpson index (F(1,16) = 0.55, *p* = 0.47) (Table 2) (Figure 2).

Similarly, richness-based metrics showed no significant differences between populations after adjustment for sequencing depth (observed richness: F(1,16) = 1.56, *p* = 0.23; Chao1 richness: F(1,16) = 1.56, *p* = 0.23), while sequencing depth remained a significant predictor of both observed richness and Chao1 (*p* = 0.044 for both). Pielou’s evenness did not differ significantly between populations (F(1,17) = 0.16, *p* = 0.70), and sequencing depth was not associated with evenness.

Sensitivity analyses based on rarefaction to an even sequencing depth (18,771 reads) yielded consistent results, with no significant population-level differences observed for entropy-based diversity, richness-based metrics, or evenness (all *p* > 0.43).

### 3.3. Beta Diversity

**Figure 3:**
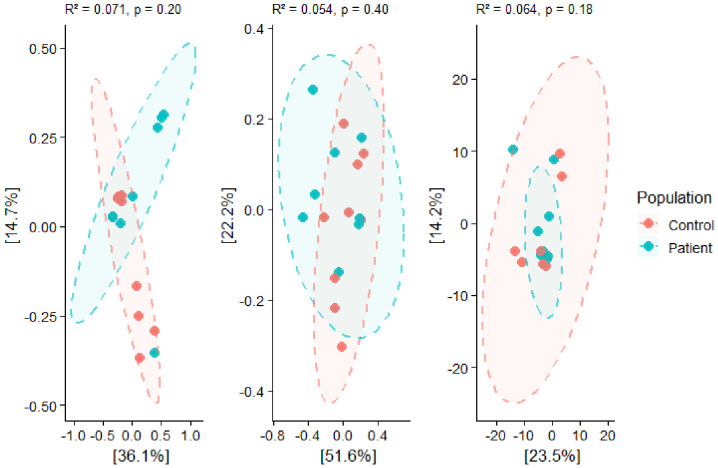
Beta diversity of the urinary microbiome in controls and patients. Principal coordinates analysis (PCoA) based on Bray–Curtis dissimilarity (relative abundance; (**left**)), weighted UniFrac distance (**middle**), and CLR–Euclidean distance (Aitchison; (**right**)). Points represent individual samples, and shaded ellipses indicate 95% confidence regions. PERMANOVA revealed no significant differences in overall community composition between controls and patients for any distance metric (Bray–Curtis: R^2^ = 0.071, *p* = 0.20; weighted UniFrac: R^2^ = 0.054, *p* = 0.40; CLR–Euclidean: R^2^ = 0.064, *p* = 0.18). Homogeneity of multivariate dispersion did not differ between groups (PERMDISP *p* > 0.35).

Beta-diversity metrics were calculated to examine whether overall urinary microbial community composition differed (Table 2). Principal coordinates analyses based on Bray–Curtis, CLR–Euclidean, and weighted UniFrac distances showed substantial overlap between controls and patients, with no clear separation of groups (Figure 3).

PERMANOVA did not detect significant differences in overall urinary microbiome composition between populations for any distance metric (Bray–Curtis: R^2^ = 0.071, F = 1.31, *p* = 0.20; CLR–Euclidean: R^2^ = 0.064, F = 1.17, *p* = 0.18; weighted UniFrac: R^2^ = 0.054, F = 0.96, *p* = 0.40).

Sensitivity analyses adjusting for log-transformed sequencing depth yielded consistent results, with neither sequencing depth nor population showing significant associations with community composition. Homogeneity of multivariate dispersions did not differ between groups for Bray–Curtis (*p* = 0.48), CLR–Euclidean distances (*p* = 0.35), or weighted UniFrac distances (*p* = 0.34), indicating that the absence of significant PERMANOVA results was not driven by differences in within-group dispersion.

### 3.4. Differential Abundance Results

Differential abundance analysis using ANCOM-BC2 did not identify any bacterial taxa that differed significantly between controls and patients after correction for multiple testing and robustness filtering. Several taxa showed nominal associations with population status prior to multiple-testing correction, but none met ANCOM-BC2 criteria for differential abundance after sensitivity and robustness checks. This result was consistent when analyses were performed at both genus level and ASV level, indicating the absence of reproducible taxon-specific differences between groups.

## 4. Discussion

The present study aimed to explore whether patients with chronic low back pain exhibit alterations in the urinary microbiome compared with pain-free controls. Across multiple complementary analytical approaches, including alpha diversity, beta diversity, and differential abundance testing, no detectable population-level differences were observed between groups in this exploratory cohort. These findings indicate that, under the present study conditions, global measures of urinary microbial diversity and community composition do not differ between individuals with chronic low back pain and pain-free controls. Baseline pain intensity was high, consistent with the clinical population referred for imaging and interventional management. Treatment efficacy was not assessed, as this study focused on urinary microbiome profiling.

Chronic pain is increasingly conceptualized as a heterogeneous, systems-level condition characterized by central sensitization, autonomic dysregulation, and persistent neuroimmune and neuroendocrine alterations [50]. Within the microbiome field, much of the existing evidence linking chronic pain to microbial dysbiosis originates from studies of the gut microbiome, where altered microbial diversity and composition have been reported across a range of chronic pain conditions [51,52,53]. The gut microbiome is uniquely positioned to reflect systemic physiological and immunological changes through its high microbial biomass [54], extensive metabolic activity [55], and direct interaction with host immune and neuroendocrine pathways [56] through the microbiota–gut–brain axis [57]. In contrast, investigations of the urinary microbiome in chronic pain remain limited and have focused predominantly on urologic chronic pelvic pain syndromes, such as interstitial cystitis/bladder pain syndrome and chronic prostatitis/chronic pelvic pain syndrome, conditions in which pain originates from, or is closely linked to, the lower urinary tract [36,58].

**Figure 4:**
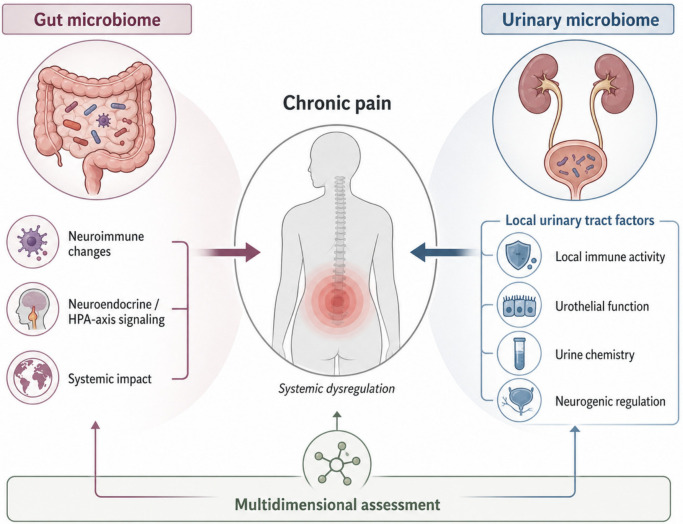
Conceptual model illustrating differential responsiveness of the gut and urinary microbiomes to chronic pain–related systemic alterations. While the gut microbiome is highly coupled to immune, neuroendocrine, and neural pathways, the urinary microbiome appears more strongly governed by local urinary tract factors, supporting its role as a complementary rather than standalone biomarker within multidimensional chronic pain frameworks.

The absence of detectable urinary microbiome differences in the present study raises the possibility that microbial alterations observed in urologic chronic pain conditions may be more closely related to local disease processes than to a generalized pain-related signature [33]. Consistent with this interpretation, the urinary microbiome is strongly shaped by proximal factors, including local immune activity, urothelial function, urine chemistry, and neurogenic regulation of bladder physiology [13,14,24]. In contrast, chronic low back pain may not impose sufficient localized perturbation on the urinary tract to induce detectable population-level shifts in microbial community structure in a small exploratory cohort, despite being associated with widespread neuroimmune and autonomic dysregulation [7,11] (Figure 4). Importantly, this interpretation does not exclude the possibility of subtle systemic host–microbe interactions, but suggests that the urinary microbiome may be less responsive to systemic pain-related alterations than intestinal microbial ecosystems under the present conditions.

While the gut microbiota has been more extensively implicated in systemic pain modulation, the present study specifically addressed the urinary microbiome, which remains underexplored in non-urological chronic pain populations. Future studies may integrate multi-site microbiome profiling, including gut and oral sites, to explore systemic interactions and correlations with urinary microbial signatures. Inflammatory biomarker profiling was beyond the scope of the present exploratory study. This limits mechanistic interpretation, as it precludes direct assessment of whether subtle immune alterations were present despite the absence of detectable urinary microbiome differences [11]. In urologic chronic pain conditions, particularly interstitial cystitis/bladder pain syndrome, urinary and tissue cytokines and chemokines have been proposed as biomarkers or mechanistic mediators of bladder inflammation and pain [18,19]. Previous studies have reported altered urinary or systemic levels of inflammatory mediators, including IL-1β, IL-6, TNF-α, IL-8/CXCL8, CXCL1, CXCL10, and CCL2/MCP-1, in patients with interstitial cystitis/bladder pain syndrome [18,19,20,21]. However, these findings cannot be directly extrapolated to non-urological chronic low back pain. Future studies should therefore combine multi-site microbiome profiling with urinary and systemic cytokine or chemokine measurements to clarify whether host–microbe–immune interactions contribute to specific chronic pain phenotypes.

An additional consideration is the inherently low-biomass and sparse nature of the urinary microbiome [30,31]. Compared with high-biomass ecosystems such as the gut, urinary microbial detection is more sensitive to sequencing depth, technical variability, and contamination [59]. In the present study, sequencing depth was associated with several diversity metrics, underscoring the importance of statistical adjustment for library size [45,47]. By incorporating sequencing depth as a covariate, performing rarefaction-based sensitivity analyses, and using compositional and phylogenetically informed beta-diversity metrics, we aimed to minimize technical bias and reduce the risk of false-positive findings [60,61]. The concordance of results across analytical frameworks [62] supports the robustness of the finding that no reproducible group-level differences were detectable in this cohort.

Taken together, our findings suggest that urinary microbial communities may be less sensitive to systemic neuroimmune and neuroendocrine alterations associated with chronic pain than the gut microbiome, at least under the present study conditions and in cohorts without primary lower urinary tract disease. This contrasts with the gut microbiome and highlights that different microbial ecosystems may vary substantially in their sensitivity to host physiological state [63,64]. Clinically, the absence of detectable population-level urinary microbiome differences in this exploratory cohort does not support its use as a single, system-level biomarker for chronic low back pain at this stage. Rather, these results align with contemporary views of chronic pain as a multidimensional condition that cannot be captured by a single biological readout [65]. This perspective is consistent with contemporary pain phenotyping frameworks that emphasize the integration of biological, psychological, and functional domains rather than reliance on single biomarkers [66,67]. In this context, urinary microbiome measures may still hold value as part of a broader, multimodal outcome framework, particularly in pain conditions with direct lower urinary tract involvement. Although the clinical pain phenotype was defined by routine clinical assessment and supported by positive scintigraphy at the corresponding lumbar facet joint, clinical characterization remained limited. Pain intensity was assessed using the VAS, but detailed multidimensional phenotyping, including functional disability, psychological status, neuropathic pain features, quality of life, and central sensitization-related measures, was not available. Because chronic low back pain is heterogeneous, future urinary microbiome studies should incorporate deeper multidimensional pain phenotyping to determine whether specific pain mechanisms or symptom profiles are associated with urinary microbial variation.

Several limitations warrant consideration. The present study was limited by its small sample size, which limits statistical power and generalizability, and the findings should therefore be considered exploratory and hypothesis-generating. Although no significant differences in alpha diversity, beta diversity, or differential abundance were detected between patients with chronic low back pain and pain-free controls, the study was not powered to exclude modest or subtle microbiome differences. Consequently, a type II error cannot be ruled out, and small but biologically meaningful alterations in urinary microbial community structure may have remained undetected. Larger, adequately powered studies are required before firm conclusions can be drawn regarding the absence of urinary microbiome alterations in non-urological chronic pain. Additionally, urine samples were collected via midstream voiding, which captures a composite signal that may include contributions from the urethra and periurethral region. More invasive sampling strategies, such as catheterized urine or paired culture-based approaches, may offer increased resolution of bladder-associated microbial communities but are less feasible in larger or non-clinical cohorts and less comfortable for participants.

Although patients and controls were matched for age and sex, several potentially relevant microbiome-related confounders were not systematically collected or controlled for. These include dietary habits, BMI, smoking status, physical activity, menopausal status, and hydration. Medication use was heterogeneous in the patient group, with participants receiving a range of analgesics and other commonly prescribed medications. Several of these drug classes have been reported to influence host physiology and may potentially affect microbial community composition. Given the exploratory study design and limited sample size, systematic adjustment for medication effects was not feasible and should be considered when interpreting the present findings. Furthermore, psychological variables and quality-of-life measures were not assessed, which may influence both pain perception and host physiological regulation. Finally, although participants with known urinary tract infection were excluded, systematic screening for asymptomatic bacteriuria was not performed, which may represent an additional unmeasured confounder.

## 5. Conclusions

This exploratory study did not identify detectable differences in urinary microbiome diversity, community composition, or taxon-specific abundance between patients with chronic low back pain and pain-free controls. These findings suggest that robust urinary microbiome alterations are not evident under the present study conditions, although larger and more deeply phenotyped studies are required to determine whether more subtle or phenotype-specific microbial signatures may exist.

## Figures and Tables

**Table 1:** Demographics for the patient group.

ID	Sex	Age (Years)	Concomitant Pharmaceutical Treatments	Pain Score
ID1	Female	50	Topiramate; sumatriptan; paracetamol; dexketoprofen + tramadol; pregabalin; pramipexole; amitriptyline; rupatadine; paroxetine	72
ID2	Female	33	Ibuprofen; paracetamol	46
ID3	Female	81	Bisoprolol; epoetin alfa; atorvastatin; olmesartan/amlodipine/hydrochlorothiazide; duloxetine; tramadol	48
ID4	Female	52	Diclofenac; oxycodone; paracetamol	72
ID5	Male	46	Celecoxib; diazepam; paracetamol; tramadol; amitriptyline; esomeprazole; semaglutide	74
ID6	Male	89	Apixaban; dapagliflozin; levothyroxine; dutasteride/tamsulosin; bumetanide; spironolactone; tafamidis	73
ID7	Female	71	Paracetamol + codeine; meloxicam; clonazepam; amitriptyline; diazepam	85
ID8	Female	73	Paracetamol + codeine	81
ID9	Female	42	Diazepam; paracetamol; tramadol; diclofenac; ethinylestradiol/levonorgestrel; folic acid	34
ID10	Male	62	/	94

Pain scores are reported on a scale ranging from 0 to 100. A slash (/) indicates that no analgesic medication was reported. All patients reported pain for ≥3 months and had a clinical diagnosis of nociceptive facet-mediated pain.

**Table 2:** Alpha- and beta-diversity of the urinary microbiome in controls and patients.

Measure	Control	Patient				
Alpha-diversity						
Observed ASVs (rarefied)	53 (21–66)	52 (24–58)				
Chao1 (rarefied)	64 (22–68)	53 (25–58)				
Shannon	1.63 (1.02–2.23)	1.26 (0.66–1.88)				
Simpson	0.698 (0.529–0.781)	0.527 (0.300–0.731)				
Inverse Simpson	3.43 (2.13–4.58)	2.11 (1.43–3.72)				
Evenness	0.42 (0.32–0.53)	0.39 (0.18–0.46)				
Beta-diversity						
Distance metric	Term	Df	Sum of squares	R^2^	F	*p*-value
Bray–Curtis	Population	1	0.359	0.071	1.31	0.20
	Residuals	17	4.667	0.929		
	Total	18	5.027	1.000		
CLR–Euclidean	Population	1	807.6	0.064	1.17	0.18
	Residuals	17	11,724	0.936		
	Total		12,531	1.000		
Weighted UniFrac	Population	1	0.0857	0.054	0.96	0.40
	Residuals	17	1.515	0.946		
	Total		1.601	1.000		

Alpha-diversity metrics are reported as median with first and third quartiles. Observed ASV richness and Chao1 are reported from rarefied data; Shannon, Simpson, inverse Simpson, and evenness were calculated from non-rarefied data. Between-sample community dissimilarity was assessed using Bray–Curtis dissimilarity on relative-abundance ASV tables, Euclidean distance after centred log-ratio (CLR) transformation, and weighted UniFrac distance. Group-level differences were tested using PERMANOVA (adonis2) with 9999 permutations. No significant differences between controls and patients were observed for any distance metric. Homogeneity of dispersions was confirmed using PERMDISP.

## Data Availability

The study involves human participants, and the urinary microbiota sequencing data contain potentially identifiable information. For this reason, the data are not publicly available without access restrictions. De-identified data may be made available for reuse under controlled access for research purposes consistent with the original study aims, following review of a data access request. Requests for data access should include a brief research proposal, intended use of the data, and confirmation of compliance with applicable data protection regulations. Requests will be evaluated by a data access committee comprising representatives of the study team and the Vrije Universiteit Brussel/UZ Brussel, and data sharing will occur in accordance with institutional and ethical guidelines. Where appropriate, access may be facilitated via a controlled-access repository.
